# Estimation of the genome sizes of the chigger mites *Leptotrombidium pallidum* and *Leptotrombidium scutellare* based on quantitative PCR and k-mer analysis

**DOI:** 10.1186/1756-3305-7-279

**Published:** 2014-06-20

**Authors:** Ju Hyeon Kim, Jong Yul Roh, Deok Ho Kwon, Young Ho Kim, Kyungjae A Yoon, Seungil Yoo, Seung-Jae Noh, Junhyung Park, E-hyun Shin, Mi-Yeoun Park, Si Hyeock Lee

**Affiliations:** 1 Department of Agricultural Biotechnology, Seoul National University, Seoul 151-921, Korea; 2 Division of Medical Entomology, National Institute of Health, Osong 363-951, Korea; 3 Research Institute of Agriculture and Life Sciences, Seoul National University, Seoul 151-921, Korea; 4 Deparment of Research, Codes Division, Insilicogen, Inc, Suwon 441-813, Korea

**Keywords:** *Leptotrombidium pallidum*, *Leptotrombidium scutellare*, Genome size, Scrub typhus, Rickettsiosis

## Abstract

**Background:**

*Leptotrombidium pallidum* and *Leptotrombidium scutellare* are the major vector mites for *Orientia tsutsugamushi*, the causative agent of scrub typhus. Before these organisms can be subjected to whole-genome sequencing, it is necessary to estimate their genome sizes to obtain basic information for establishing the strategies that should be used for genome sequencing and assembly.

**Method:**

The genome sizes of *L. pallidum* and *L. scutellare* were estimated by a method based on quantitative real-time PCR. In addition, a k-mer analysis of the whole-genome sequences obtained through Illumina sequencing was conducted to verify the mutual compatibility and reliability of the results.

**Results:**

The genome sizes estimated using qPCR were 191 ± 7 Mb for *L. pallidum* and 262 ± 13 Mb for *L. scutellare*. The k-mer analysis-based genome lengths were estimated to be 175 Mb for *L. pallidum* and 286 Mb for *L. scutellare*. The estimates from these two independent methods were mutually complementary and within a similar range to those of other Acariform mites.

**Conclusions:**

The estimation method based on qPCR appears to be a useful alternative when the standard methods, such as flow cytometry, are impractical. The relatively small estimated genome sizes should facilitate whole-genome analysis, which could contribute to our understanding of Arachnida genome evolution and provide key information for scrub typhus prevention and mite vector competence.

## Background

Scrub typhus, which is also known as chigger-borne rickettsiosis, is an acute, febrile disease caused by an obligate intracellular bacterium, *Orientia tsutsugamushi*. This pathogen causes fever, rash, eschar formation and pneumonitis and the symptoms can vary from mild to fatal [1]. If not treated with appropriate antibiotics, mortality rates by scrub typhus are reported to be in 1% to 40%, depending on area and *O. tsutsugamushi* strain [2]. This disease is distributed extensively from the Asia-Pacific region, including Japan, Korea, China, India, Pakistan, the southwestern Pacific islands, and Australia, to the eastern part of Russia [3]. More than one million cases of scrub typhus are reported every year and more than one billion people are exposed to the risk of infection [4].

Humans acquire the disease through a bite from infected larval mites of the family Trombiculidae known as chiggers. Trombiculid mites are very small (0.2 - 0.4 mm) and usually inhabit grassy and weedy areas. Chigger is the only parasitic life stage that bites hosts and transmits the disease because other stages, such as nymph and adult, do not feed on hosts [5]. Vertebrate animals, mostly ground-dwelling rodents, are the natural hosts for chiggers, whereas humans are an incidental host. The chigger pierces the host’s skin with its sharp mouthparts and injects digestive enzymes, thereby dissolving tissues of the host for ingestion [5]. Among trombiculid mites, some species belonging to the genus *Leptotrombidium* are known to transmit *O. tsutsugamushi*[6], but the major vector species differs from country to country. *Leptotrombidium pallidum* is widely distributed in Korea [7,8], Japan [3,9-11], and Russia [3,11] and is the primary vector mite in the Korean Peninsula [8,12,13] and Japan [1,14]. *Leptotrombidium scutellare*, which is also distributed in Korea [7,8], Japan [3,9-11], China [15], Thailand [3,16], and Malaysia [3,17], serves as the predominant transmission vector in the southern part of Korea [8,13], Japan [1,14,18], and northern China [15]. Recent studies have reported that *Leptotrombidium* mites carry other pathogens, such as hantavirus [19] and *Bartonella tamiae*, the causative agent of human bartonellosis [20], suggesting that these mites may serve as vectors for a wider variety of pathogens than has been commonly expected. Despite their medical importance, little molecular information on *Leptotrombidium* mites is available to date. In addition, the molecular and genetic bases of their vector competence are unknown, and there are no available efficient methods for their control.

The whole-genome sequencing of the *Leptotrombidium* mite would provide fundamental genetic information for understanding vector competence, discovering new target sites for novel acaricides and repellents, and eventually designing efficient measures to prevent scrub typhus. In addition to the completed genomes of *Homo sapiens*[21] as the host and *O. tsutsugamushi*[22] as the pathogen, genomic information of *Leptotrombidium* mite vectors would enable an understanding of the mite vector-host-pathogen interactions. Comparative genomic and transcriptomic analyses between the two *Leptotrombidium* species would eventually provide basic information on how the differences in their genomic (or transcriptomic) components contribute to the phenotypic differences (i.e., biological and ecological differences) and on what conserved molecular genetic natures are commonly responsible for their vector competence. Prior to the genome sequencing of *L. pallidum* and *L. scutellare*, however, an accurate estimation of their genome size is necessary to ensure sufficient sequencing coverage, particularly if the genome sequencing is conducted through next-generation sequencing (NGS) methods, and to provide a firm reference for genome assembly.

Although flow cytometry is regarded as a standard method for the prediction of the genome size of multicellular organisms [23,24], it is not applicable to all arthropods, particularly if the body size is too small to obtain a sufficient amount of genetic material or if it is difficult to obtain a sufficient number of cells from the body preparations [25,26]. As an alternative, a method for the estimation of the genome size based on quantitative real-time PCR (qPCR) was developed [27] and was determined to be reliable and useful for predicting the genome sizes of several arthropods, including *Musca domestica*[28], *Metaseiulus occidentalis*[26], *Sarcoptes scabiei*, *Psoroptes ovis*, *Dermatophagoides pteronyssinus*[25], and *Cotesia plutellae*[29].

In this study, the genome sizes of *L. pallidum* and *L. scutellare,* as vector mites of scrub typhus, were estimated by qPCR. To examine the validity and accuracy of the method, we used three arthropods with their genome analysis completed, namely *Drosophila melanogaster*, *Apis mellifera*, and *Tetranychus urticae,* as internal references. In addition, the genome size was also estimated through the k-mer analysis of the Illumina sequencing reads to mutually confirm the results.

## Methods

### *L. pallidum* and *L. scutellare*

To collect *L. pallidum*, Sharman traps baited with crackers and peanut butter were set for wild black-striped mice, *Apodemus agrarius*, on grassy areas near a stream in Cheorwon-gun, Gangwon province, South Korea. The traps were laid out in the late afternoon and retrieved the following morning. All of the ectoparasites on the collected mice were harvested, *L. pallidum* was identified and isolated through microscopic inspection. The collected *L. palladium* larvae were directly used for genomic DNA isolation. The black-striped mice were captured and handled based on ethical procedures and scientific care according to the animal use protocol that had been reviewed and approved by the Korea Center for Disease Control & Prevention-Institutional Animal Care and Use Committee (KCDC-IACUC; KCDC-046-13-2A).

Laboratory strains of *L. pallidum* and *L. scutellare* have been maintained for eight generations and one generation, respectively, in the rearing facility of the Korea Centers for Disease Control and Prevention, Osong, Korea. Both *L. pallidum* and *L. scutellare* have been reared in rearing chambers containing a charcoal-plaster mixture (calcium sulfate hemihydrate and charcoal powder, 9:1) and fed eggs of *Sinella curviseta*.

### Cloning of single copy genes

The total RNA from nine *L. pallidum* and five *L. scutellare* females reared in the laboratory was extracted using 100 μl of TRI reagent (MRC, Cincinnati, OH, USA) according to the manufacturer’s protocol. The first-strand cDNA was synthesized from the DNase I (Takara, Japan)-treated total RNA using SuperScript III reverse transcriptase (Invitrogen, Carlsbad, CA, USA) and used as the PCR template. The degenerate primers were designed from conserved amino acid regions of two putative single copy genes, namely elongation factor 1 α (EF1 α) and ribosomal protein S3 (RpS3), across various mite species. The primer sets used for the PCR analysis are provided in Table 1. The PCR was conducted in a DNA Engine Peltier Thermal Cycler (Bio-Rad, Richmond, CA, USA) using the following cycling conditions: a single denaturation cycle at 95°C for 2 min and 35 cycles of 95°C for 15 s, 55°C (EF1 α) or 45°C (RpS3) for 20 s, and 68°C for 1 min. The PCR products of appropriate sizes were excised from agarose gels, purified with a QIAquick PCR Purification Kit (Qiagen, Valencia, CA, USA), and then cloned into the pGEM-T easy vector (Promega, Madison, WI, USA).

**Table 1:** Primers used in this study

**Purpose**	**Species**	**Gene**	**Sequence (5’ to 3’)**		**Product size (bp)**
Cloning	*Leptotrombidium pallidum* *Leptotrombidium scutellare*	EF1α	F^a^	TATTGATGCTCCTGGTCACAG	850
			R^b^	GAATTTGCAAGCAATGTGAGC	
		RpS3	F	GARGAYGGNTAYTCHGGB	438
			R	CATRAYYTTNACYTTRATDCC	
Standard	*Drosophila melanogaster*	RpS3	F	CACGTTTCCGATTCGACGTC	925
			R	CACAACGGACACATTGTCGG	
	*Apis mellifera*	RpS3	F	CTCGTGAACTGTCAGAAGATG	794
			R	CTGCAAGTGGTATTGGTTGTG	
	*Tetranychus urticae*	RpS3	F	TAGACGAATTCCTTCGTCGAG	553
			R	AGAAACGTTGTCAGGTAATGGT	
	*Leptotrombidium pallidum* *Leptotrombidium scutellare*	EF1α	F	TTGATGCTCCTGGTCACAGA	842
			R	GCAAGCAATGTGAGCAGTGT	
		RpS3	F	ATCATCATTCTCGCCACGCG	447
			R	CTTGTCGCAGTAACACATGCC	
qPCR	*Drosophila melanogaster*	RpS3	F	CATTGAGTTGTACGCCGAGA	127
			R	ATGTAGCGGAGCACACCATAG	
	*Apis mellifera*	RpS3	F	GTTGTGAAGTGGTTGTTAGTG	132
			R	GAAGTACATGACGAGTTGCAG	
	*Tetranychus urticae*	RpS3	F	ATGTGAAGTTGTCGTTTCCGG	96
			R	TTACAAGGGTCACCAGCGTG	
	*Leptotrombidium pallidum*	EF1α	F	GTTAAGGAATTGCGCAGAGG	123
			R	GTAACCGTTGGCGATTTGTC	
		RpS3	F	TCTGACAGAGGCTTGTGTGC	127
			R	AGCCTTTCGCTCCAGATTCC	
	*Leptotrombidium scutellare*	EF1α	F	CCGGAGATTGGAACGAAAGG	120
			R	TGGACACAACTGAACCACCC	
		RpS3	F	GCACAATGCGAGTCTCTTCG	111
			R	GACTTCGCAACCTTTCGCTC	

^a^Forward primer.

^b^Reverse primer.

### Extraction of genomic DNA

The genomic DNA (gDNA) from 170 *L. pallidum* larvae and 15 *L. scutellare* female adults was extracted and used for qPCR. The gDNA from 10 female *D. melanogaster*, a single worker *A. mellifera*, and 100 female *T. urticae* were also extracted and used as internal controls. The DNA extraction was performed using the Qiagen DNeasy Blood and Tissue Kit (Qiagen, Valencia, CA, USA) according to the manufacturer’s instructions. After extraction, the gDNA was treated with 20 μl of proteinase K (Qiagen, 0.5 mg/ml) and 2 μl of RNase A (Qiagen, 0.2 mg/ml) to remove any protein and RNA contamination, respectively. The DNA was eluted with 10 mM Tris and 0.1 mM EDTA buffer (pH 8.5), and aliquots were stored at -20°C. The quality and concentration of the gDNA was determined using a NanoDrop spectrophotometer (NanoDrop Technologies, Wilmington, DE, USA) and by agarose gel electrophoresis using a mass ladder (Invitrogen, Carlsbad, CA, USA).

### Preparation of standard DNA

The gDNA fragments of EF1α and RpS3 for *L. pallidum* and *L. scutellare* and of RpS3 for *D. melanogaster*, *A. mellifera*, and *T. urticae* were generated by PCR using the extracted gDNA samples as templates and individual primer sets (Table 1). The PCR assays were conducted with 0.5 μM forward and reverse primers, 250 μM dNTPs, 10 ng gDNA template, and 1 U of Advantage 2 DNA polymerase mix (Clontech, Palo Alto, CA, USA) in a total volume of 20 μl. The PCR protocol consisted of an initial denaturation step of 95°C for 2 min followed by 35 cycles of 95°C for 15 s, 55°C (EF1 α) or 45°C (RpS3) for 20 s, and 68°C for 1 min. The PCR products were confirmed by agarose gel electrophoresis, purified using a QIAquick Gel Extraction Kit (Qiagen, Valencia, CA, USA), and then cloned into the pGEM-T easy vector (Promega, Madison, WI, USA). The positive plasmids confirmed by sequencing were linearized with SalI (Koschem, Seoul, Korea), purified, and quantified using the same method described above. Seven serial dilutions of the linearized plasmids ranging from 200 pg/μl to 0.2 fg/μl were produced for standard DNA preparation.

### qPCR

The quantity of the target gene in the gDNA was estimated using the qPCR method. The amplification reactions contained 0.5 μM nested primer pairs (Table 1), the DyNAmo HS SYBR Green master mix (Finnzyme, Espoo, Finland), and 15–25 ng gDNA or 5 μl of the serially diluted standard DNA. The qPCR assays were performed using an Opticon 3 thermal cycler (MJ Research, Waltham, MA, USA) with the following program: 95°C for 15 min, 40 cycles of 95°C for 10 s, 58°C for 20 s, and 72°C for 30 s. The melting curve analysis was conducted by serially increasing the temperature at a rate of 0.2°C per 1 s from 45°C to 95°C. The copy number of each standard DNA sample was calculated from the amount and molecular mass of the linearized plasmid using a DNA molecular weight calculator (http://www.currentprotocols.com/WileyCDA/CurPro3Tool/toolId-8.html). The Ct values were determined using the Opticon Monitor Software (MJ Research). The standard curve of the Ct value vs. the copy number was generated and used to calculate the total number of genome in the target gDNA template. The experiments were repeated six times, and each repetition included two technical replicates.

### Calculation of the genome sizes

The genome size was estimated using two different formulas: (1) genome size (bp) = C_A_ × B^-1^, where C_A_ is the mass of a single genome in picograms and B is the mean mass of one nucleotide base pair (1.023 × 10^-9^ pg) [30], and (2) genome size (bp) = C_B_ × N_A_ × M_Bp_^-1^, where C_B_ is the mass of a single genome in grams, N_A_ is Avogadro’s number, and M_Bp_ is the mean molar mass of one nucleotide base pair (660 g/mol) [27]. The genome size estimates were determined by averaging the values obtained from the formula.

### k-mer analysis

NGS libraries with an insert size of 350 bp were separately prepared from 150 ng gDNA of *L. pallidum* and *L. scutellare* using the TruSeq Nano DNA Sample Prep Kit (Illumina Inc., San Diego, CA, USA) following manufacturer’s standard protocols. One lane of paired-end sequencing (2x101 bp) for each organism was performed using the HiSeq2000 platform (Illumina Inc.), which produced 357,940,882 raw sequence reads for *L. pallidum* and 347,063,430 reads for *L. scutellare*. These raw sequence reads were subjected to pre-processing using CLC Assembly Cell (CLCBio, Arhaus, Denmark), during which the reads with a low quality score (less than Q20) were trimmed and the reads derived from duplicates or bacterial contaminations were removed.

The genome size estimation based on the k-mer frequency distribution was basically implemented as described previously (see supplementary information of [31]). In brief, 25,822,367,784 bp of high-quality reads for *L. pallidum* and 30,090,908,545 bp for *L. scutellare* were subjected to k-mer counting using the JellyFish program [32] with a k-mer size of 17. The k-mer frequency distribution curve was plotted with the k-mer depth as the x-axis and the k-mer frequency as the y-axis. The genome coverage depth was calculated using following formula:

Genome coverage depth = k-mer coverage depth × average read length × (average read length - k-mer size + 1)^-1^, where the k-mer coverage depth is the maximal peak in the curve. The genome size was then estimated as follows:

$$\text{Genome}\;\text{size}=\text{total}\;\text{base}\;\text{number}\times {\left(\text{genome}\;\text{coverage}\;\text{depth}\right)}^{-1}$$

## Results and discussion

### Single-copy gene cloning and standard preparation

The PCR amplification of the cDNA from *L. pallidum* and *L. scutellare* using degenerate primers for EF1α (Table 1) yielded DNA products approximately 850 bp in size. The products were cloned, and 850-bp and 841-bp cDNA sequences were obtained from *L. pallidum* and *L. scutellare*, respectively. A BLAST search of the GenBank database using the deduced amino acid sequences as queries confirmed that the sequences were putative partial sequences of the EF1α gene. The comparison of the partial sequences between *L. pallidum* and *L. scutellare* displayed 95.8% and 98.9% identities in the nucleotide and amino acid sequences, respectively. The comparison of the deduced amino acid sequences with those of *T. urticae*, which belongs to the same order, i.e., Trombidiformes, revealed that *L. pallidum* and *L. scutellare* exhibit 87.5% and 86.2% identities, respectively. The partial gDNA fragments of *L. pallidum* (842 bp) and *L. scutellare* (843 bp) were amplified with gene-specific primers designed from the cDNA sequences for standard DNA preparation, and the fragments did not contain any introns.

The 438-bp and 398-bp RpS3 cDNA fragments from *L. pallidum* and *L. scutellare* were obtained using degenerate primers. A BLAST search using the deduced amino acid sequences showed that the partial sequences have functional domains of RpS3. The amino acid sequences of the two *Leptotrombidium* species were equal and showed 94.5% identity when compared with that of *T. urticae*. The 448-bp gDNA fragments containing a 50-bp intron were amplified from both *L. pallidum* and *L. scutellare* for standard DNA preparation. For comparison to reference arthropods, the respective gDNA fragments of RpS3 gene (925 bp for *D. melanogaster*, 793 bp for *A. mellifera*, and 629 bp for *T. urticae*) were obtained and cloned for standard DNA preparation.

### Estimation of the genome size

The estimation of the genome sizes of the reference arthropods by qPCR using RpS3 as the target gene revealed estimated sizes of 188 ± 9 Mb, 283 ± 14 Mb, and 89 ± 4 Mb for *D. melanogaster*, *A. mellifera*, and *T. urticae*, respectively (Table 2). These estimates were similar to the published values for the actual genome sizes (180 Mb for *D. melanogaster*[33], 236 Mb for *A. mellifera*[34], and 90 Mb for *T. urticae*[35]), showing the high prediction accuracy (79.8 – 98.9%) and reliability of the qPCR method for genome size prediction.

**Table 2:** Genome sizes of the reference arthropods estimated by the qPCR-based method

**Species**	**Gene**	**Actual size (Mb)**^**a**^	**Estimated size (Mb)**			**Estimation accuracy (%)**
			**Formula from Dolezel *et al*.**^**b**^	**Formula from Wilhelm *et al.***^**b**^	**Average**	
*Drosophila melanogaster*	RpS3	180	182 ± 14	195 ± 15	**188 ± 9**	95.1
*Apis mellifera*	RpS3	236	273 ± 86	293 ± 92	**283 ± 14**	79.8
*Tetranychus urticae*	RpS3	90	86 ± 36	92 ± 39	**89 ± 4**	98.9

^a^Actual genome size information was obtained from references [33,34], and [35] for *D. melanogaster*, *A. mellifera* and *T. urticae,* respectively.

^b^For the formula ‘Dolezel *et al*.’ and ‘Wilhelm *et al*.’, please refer references [30] and [27], respectively.

The estimation of the genome size using qPCR with two single-copy genes (EF1α and RpS3) of *L. pallidum* revealed a genome size of 185 ± 42 Mb and 197 ± 47 Mb based on the formula described by Dolezel *et al.*[30] and Wilhelm *et al.*[27], which yielded a mean estimate of 191 ± 7 Mb (Table 3). Similarly, the genome size of *L. scutellare* was estimated to be 253 ± 22 Mb and 271 ± 24 Mb using the two different formulas, respectively, which resulted in an average size estimate of 262 ± 13 Mb (Table 3).

**Table 3:** **Genome sizes of *Leptotrombidium pallidum *** **and *Leptotrombidium scutellare *** **estimated by the qPCR and k-mer analysis-based method**

**Species**	**Method**	**Formula**^**a**^	**Estimated size (Mb)**		
			**EF1α**	**RpS3**	**Average**
*Leptotrombidium pallidum*		Dolezel *et al.*	155 ± 42	215 ± 39	185 ± 42
	qPCR	Wilhelm *et al.*	164 ± 45	231 ± 42	197 ± 47
		**Average**			**191 ± 7**
	k-mer analysis				**175**
*Leptotrombidium scutellare*		Dolezel *et al*.	269 ± 11	237 ± 20	253 ± 22
	qPCR	Wilhelm *et al*.	288 ± 12	254 ± 22	271 ± 24
		**Average**			**262 ± 13**
	k-mer analysis				**286**

^a^For the formula ‘Dolezel *et al.*’ and ‘Wilhelm *et al*.’, please refer references [30] and [27], respectively.

To confirm the estimates obtained by qPCR, the genome sizes were also estimated through a k-mer analysis of the Illumina sequencing reads. The k-mer method has been successfully applied for the estimation of the genome size from NGS reads and has provided practical guidance for the design of NGS sequencing and genome assembly for several genome projects without prior knowledge of the genome size, such as the analysis of the genomes of the giant panda [36], cucumber [37], and pacific oyster [31]. Fundamentally, k-mer analysis is based solely on the sequence contents of NGS reads. Thus, if the NGS reads well represent the whole contents of the genome without any bias during the experimental procedures, including the isolation of genomic DNA, the construction of NGS libraries, and the high-throughput sequencing steps, the k-mer output should give a close estimate of the genome size.

**Figure 1:**
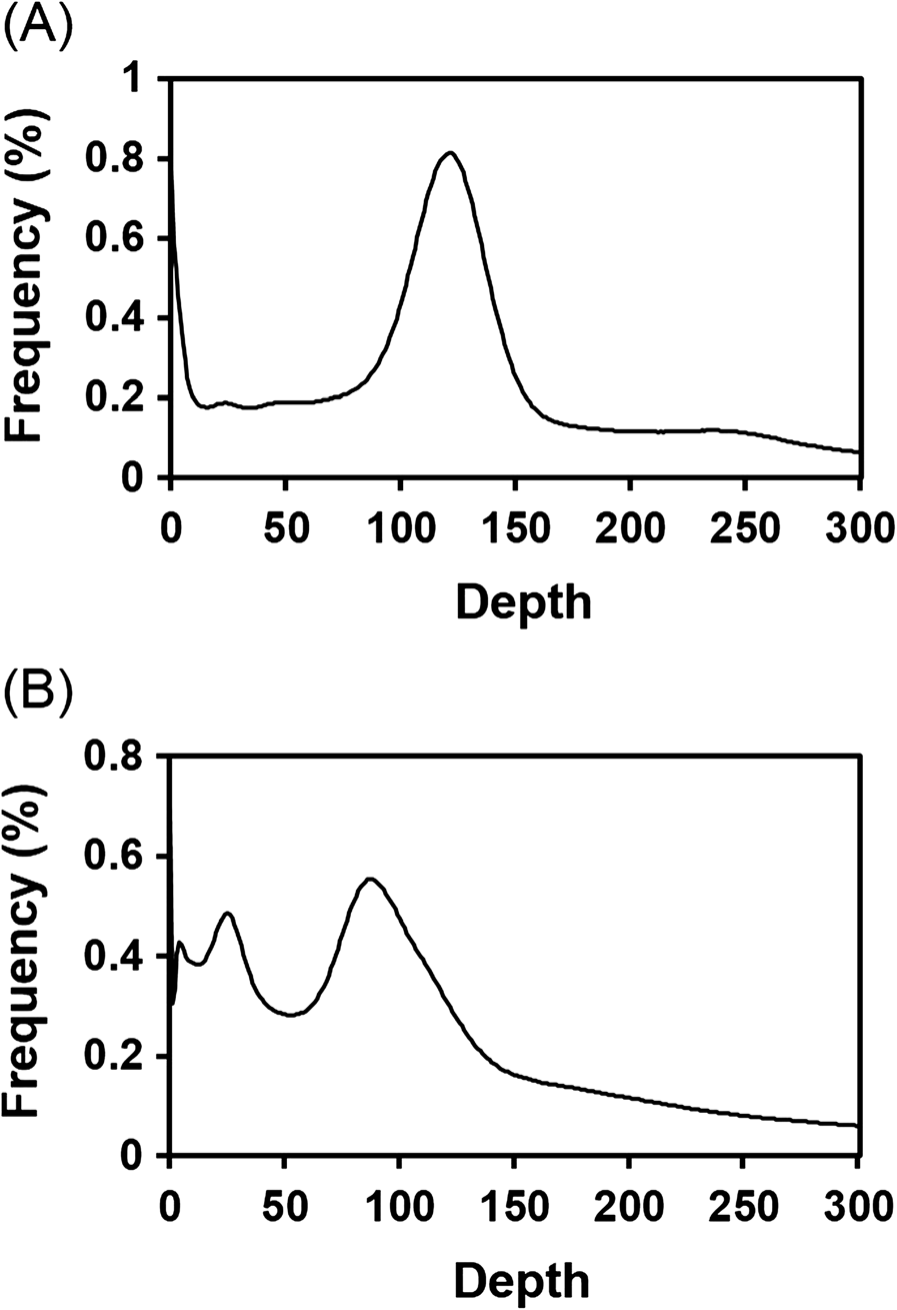
**K-mer frequency distribution curve.** All 17-mer sequences were extracted from pre-processed high-quality paired-end reads and plotted the frequency (y-axis) as a function of the depth (x-axis). **(A)** k-mer curve for *L. pallidum*. The single main peak indicates a homozygous genomic source. **(B)** k-mer curve for *L. scutellare.* Instead of a single peak, minor residual peaks are also shown, indicating the heterozygous nature of the isolated genomic material.

The resulting values obtained from the k-mer analysis were 175 Mb for *L. pallidum* and 286 Mb for *L. scutellare* (Figure 1). Compared with the values obtained by qPCR, the estimates obtained for *L. pallidum* and *L. scutellare* based on k-mer analysis were 16-Mb smaller and 24-Mb larger, respectively. The smaller *L. pallidum* genome size based on the k-mer analysis compared with the qPCR estimate (i.e., 175 Mb vs. 191 Mb) may be the result of the omission of parts of the genome, such as heterochromatic regions or highly repetitive regions, during the NGS process. In contrast, for *L. scutellare*, the k-mer estimate was larger than the qPCR estimate (i.e., 286 Mb vs. 262 Mb). The k-mer frequency curve showed the existence of minor residual peaks, which indicates that there may be genomic contamination or a certain level of heterozygosity in the genomic pool of *L. scutellare* (Figure 1). Nevertheless, the calculation of the mean values between these two methods revealed that the genome sizes of *L. pallidum* and *L. scutellare* were 183 Mb and 274 Mb, respectively. The mean deviations accounted for only 8.7% and 8.8% of the respective mean genome size estimates between the two methods, suggesting that these estimates are mutually complementary.

Interestingly, the estimated genome size of *L. scutellare* appears to be approximately 1.5-fold larger than that of *L. pallidum* even though they belong to the same *Leptotrombidium* genus. Because both species have very similar biology and ecology, this genome size difference is not likely due to a difference in the gene numbers but rather to differences in the non-coding sequences. A similar genome size difference between closely related species within the same genus was reported in the comparison of *D. melanogaster* and *D. virilis*: the euchromatic genome size of *D. virilis* (150 Mb) was 36% larger than that of *D. melanogaster* (110 Mb), and this difference was well correlated with the significant increase in the intron size [38]. Similarly, genome size differences are also found in several arthropod species within the same genus: the genome estimates of *Pseudacteon tricuspis* (746 Mb) and *P. obtusus* (613 Mb) showed a 20% difference, the estimated genome size of *Calosoma scrutator* (1,019 Mb) was 39% larger than that of *C. sayi* (732 Mb), and *Polistes exclamans* (542 Mb) has a 44% larger genome than *P. Carolina* (376 Mb) [39].

There are a variety of other factors that may result in an increase in genome size, including increases in the copy number of transposable elements, the amount of simple repeated sequences, the size of inter-enhancer spacers, the amount or size of microsatellites, and the presence of large numbers of pseudogenes (reviewed by [40]).

**Figure 2:**
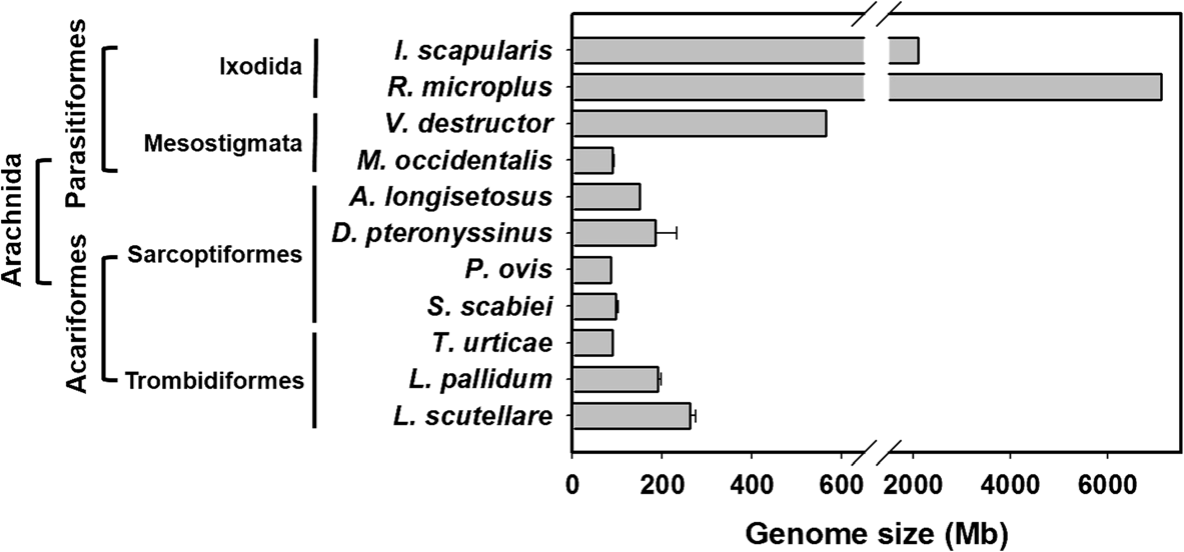
**Comparison of the genome sizes of arachnids.** The genome sizes of 11 arachnids in 4 orders, *Ixodes scapularis*[41], *Rhipicephalus microplus*[41], *Varroa destructor*[42]*, Metaseiulus occidentalis*[26], *Archegozetes longisetosus*[39], *Dermatophagoides pteronyssinus*, *Psoroptes ovis*, *Sarcoptes scabiei*[25], *Tetranychus urticae*[35], *Leptotrombidium pallidum* and *Leptotrombidium scutellare*, were compared.

The comparison of the estimated genome sizes with those of other mites or ticks revealed that the estimates are larger than those of *T. urticae* (90 Mb), which belongs to the same order (Trombidiformes), *S. scabiei* (96 ± 7 Mb) [25], and *P. ovis* (86 ± 2 Mb) [25]. However, the estimated genome sizes are similar to those of other Acariformes mites and markedly smaller than those of Parasitiformes ticks, such as *Ixodes scapularis* (2.1 Gb) and *Rhipicephalus microplus* (7.1 Gb) [41] (Figure 2). Although the C-value paradox is also applicable for the various genome sizes across the major groups of mites and ticks within the subclass Acari, the relatively smaller genome sizes of mites compared to those of true ticks appear to be positively correlated with their smaller cellular or nuclear sizes (i.e., smaller body sizes).

## Conclusions

In this study, the genome sizes of the scrub typhus vectors *L. pallidum* and *L. scutellare* were estimated using the qPCR-based calculation and k-mer analysis. The determined sizes were 183 Mb for *L. pallidum* and 274 Mb for *L. scutellare*. Although flow cytometry could not be performed due to the limited genetic material, the results from the two methods were within the same range and thus likely reliable. Such relatively small genome sizes should enable a more successful analysis of the whole genomes of these chigger mites even based on NGS, and the genome size estimates may serve as firm reference values for the genome assembly following sequencing.

Starting with the *I. scapularis* genome project, which was the first in the subphylum Chelicerata, several studies have attempted to obtain genomic information for arachnids, such as *Varroa destructor*[42], *Boophilus microplus*[43], *Metaseiulus occidentalis*[44], and *Tetranychus urticae*[35]. In addition to the previous studies, the genome sequencing of *Leptotrombidium* mites may contribute to the understanding of mite vector biology, Arachnida genome evolution, and molecular interaction between mite vector and *O. tsutsugamushi,* and eventually provide key information for developing novel strategies for scrub typhus control.

## Competing interests

The authors declare that they have no competing interests.

## Authors’ contribution

JHK, JYR, DHK, YHK, MP and SHL designed and performed the genome size estimation based on the qPCR analyses. JHK and SHL wrote the manuscript. JHK, JYR, ES and KAY maintained the laboratory strains of *L. pallidum* and *L. scutellare.* SY, S-JN and JP performed the genome size estimation using k-mer analysis. All authors read and approved the final version of the manuscript.
